# Receptionist rECognition and rEferral of PaTients with Stroke (RECEPTS) study - protocol of a mixed methods study

**DOI:** 10.1186/1471-2296-15-91

**Published:** 2014-05-12

**Authors:** James P Sheppard, Satinder Singh, Janet Jones, Elizabeth Bates, John Skelton, Connie Wiskin, Richard J McManus, Ruth M Mellor

**Affiliations:** 1 Department of Primary Care Health Sciences, University of Oxford, Oxford, UK; 2 General Practitioner, Northfield Health Centre (Tudor Practice), Birmingham, UK; 3 Primary Care Clinical Sciences, University of Birmingham, Birmingham, UK

**Keywords:** Medical receptionists, General Practice, Family practice, Health Services Administration, Simulation, Patient, Research, Qualitative, Questionnaires

## Abstract

**Background:**

As the first point of contact for patients and witnesses of stroke, General Practice receptionists can be instrumental in deciding the urgency of clinical contact. Despite the considerable complexity of this task, reception staff are not clinically trained. Minimising the time taken to access thrombolysis is crucial in acute stroke as treatment must be initiated within 4.5 hours of the onset, and the earlier the better, to achieve the best outcomes. Research suggests that patients who first contact their General Practice following the onset of stroke symptoms are less likely to receive thrombolysis, in part due to significant delays within Primary Care.

This study therefore aims to understand the role of General Practice receptionists, with particular interest in receptionist’s ability to recognise people who may be suffering from a stroke and to handle such patients as a medical emergency.

**Methods:**

The Receptionist rECognition and rEferral of PaTients with Stroke (RECEPTS) study will be a Primary Care based mixed methods study. 60 General Practices in the West Midlands will be recruited. Each practice will receive 10 unannounced simulated patient telephone calls, after the 10 calls questionnaires will be administered to each receptionist. These will examine the behaviour of receptionists towards patients presenting in Primary Care with stroke symptoms, and their knowledge of stroke symptoms. An embedded qualitative study will use interviews and focus groups to investigate the views of General Practice staff on the receptionists’ role in patient referral and whether training in this area would be helpful.

**Discussion:**

The results of the RECEPTS study will have important implications for providers of Primary Care. The study will establish current practice in UK primary care in terms of General Practice receptionists’ knowledge of the presentation and appropriate referral of those who may be suffering a stroke. It will highlight training needs and how such training might be best delivered.

## Background

In the UK, as in similar contexts, General Practice (GP) receptionists operate in the interface between patients and their doctors. They are usually the first person a patient, spouse or other witness of an event, speaks to when contacting their GP and they are instrumental in deciding on the urgency of clinical contact and when (or if) an appointment will be made [1,2]. As such they have an important gate-keeping role [3] and are influential in the facilitation of the patient journey.

Despite being integral members of the Primary Care Team, GP reception staff may be minimally trained, often learning about their role from other receptionists while on-the-job [4]. This may impair the ability of reception staff to correctly identify the level of urgency required from patients seeking access to healthcare which is increasingly important as new acute treatments are developed in a range of different illnesses. The need for appropriate training has been consistently recognised by researchers, doctors and reception staff themselves [5-7].

In the UK, there are 152,000 new stroke cases every year [8]. The direct economic costs to the NHS are considerable, accounting for approximately 5% of total UK NHS costs or £4 billion [9]. The burden of stroke can be considerably reduced if patients are rapidly thombolysed following the onset of symptoms by specialists in secondary care [10], as soon as possible after symptom onset [11], and within the 4.5 hours ‘therapeutic window’ [12-14]. Yet only 4-5% of patients with stroke receive thrombolysis in developed countries [15,16]. A significant barrier to thrombolysis is the time delay between patients experiencing symptoms of a stroke, contacting healthcare services [17] and correctly being identified and treated by healthcare staff [18]. Fewer than half patients reported recognising their own stroke [19] and members of the public vary greatly in the their knowledge of stroke symptoms [20]. Patients and bystanders help seeking behaviour can be influenced by perception of the severity of the symptoms [21-23], how they fitted the symptoms into their normal life [23], the influence of others [21,23] and concern around the consequences of contacting medical services [22].

Previous studies suggest that contacting the GP following the onset of stroke symptoms is one such cause of thrombolysis time delay [24]. Indeed, it is estimated that only 55-71% of patients who call their GP with symptoms of stroke are correctly referred on to the emergency services [24,25]. However, these existing studies are limited due to small sample sizes and the methodology used to obtain these estimates.

### Study aims

This study aims to:

1. Assess receptionists’ ability to direct patients with, or witnesses of, stroke symptoms for emergency care, and their knowledge of stroke symptoms.

2. Investigate the views of Primary Care staff on the receptionists’ role in relation to patient access to emergency care.

## Methods

### Study design

**Figure 1:**
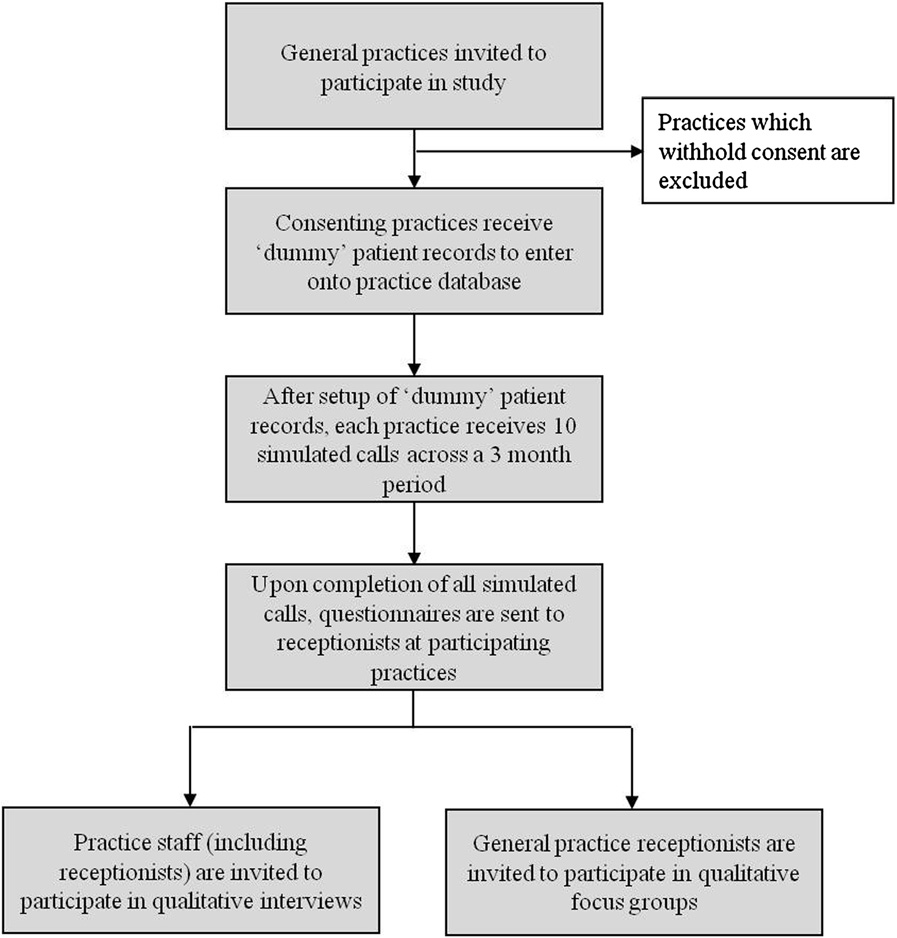
Process of recruitment and data collection during the study.

The project will be a Primary Care based mixed methods study. Assessment will be carried out using unannounced simulated patient telephone calls, questionnaires, an answerphone assessment and qualitative interviews and focus groups; the setting and data collected by these methods is summarised in Table 1. The sequence of these methods is outlined in Figure 1. Unannounced simulated patient telephone calls and answerphone assessments will be conducted prior to receptionists receiving questionnaires or invitations to participate in a focus group. Non-reception GP staff can be invited for interview at any point in the study, as their involvement in an interview should not influence simulated call results.

**Table 1:** Summary of RECEPTS methods, setting and data collected

**Setting**	**Method**	**Data collected**
UoB (telephone call to General Practice)	Unannounced simulated patient telephone call	Receptionists response to stroke symptoms
UoB (telephone call to General Practice)	Voice recording	Record the General Practice out-of-hours message
General Practice	Questionnaire (anonymous)	Receptionist demographic data.
		Receptionist knowledge and planned response to stroke and a variety of other symptoms
General Practice	Focus Groups	Receptionist views on their role in the triage of patients and their views on training for receptionists
General Practice	Interviews	The views of other Primary Care staff on the role, skills and potential of reception staff

UoB, University of Birmingham.

### Study population

The population of interest are reception staff based in collaborating GPs in NHS Primary Care Providers within Birmingham and Solihull, West Midlands, UK. All practices within this region will be invited to participate and those agreeing will provide written practice level consent, from both a General Practitioner and the Practice Manager (or equivalent). Receptionists within participating practices will be informed about the study by the Practice Manager but they will not be told the calls will be specifically about stroke. For the questionnaires, consent will be assumed by their completion. Separate individual written consent will be obtained for those taking part in interviews and focus groups.

All participating practices will be reimbursed for any additional work required to participate in the study.

### Unannounced simulated patient telephone calls

Medical role players will make telephone calls to GPs using vignettes of patients experiencing stroke-like symptoms to guide their performance. Simulated patient scenarios are an accepted methodology for assessing clinical performance, particularly in Primary Care [26,27]. This methodology has been used for a variety of clinical performance evaluation studies [28-30] including telephone triage of chest pain patients for immediate hospital care [31,32]. However, despite the context, content and face validity of unannounced simulated patient telephone calls in Primary Care, its use among GP reception staff is novel and untested in the literature. The Interactive Studies Unit (at the University of Birmingham) has successfully supported medical receptionist training for a number of years. Role players taking part in the study will all be members of the Interactive Studies Unit trained specifically for teaching, research and assessment purposes in a medical education context.

Ten unannounced simulated patient telephone calls made during the normal working day will be conducted at each practice over a three month period. Ten different stroke vignettes will be delivered, designed to represent the wide range of presentations of stroke that could be encountered by reception staff in Primary Care. The vignettes were designed by the team, including two General Practitioners for clinical input, stroke patient representatives were consulted regarding specific phrasing of the vignettes and role players reviewed the vignettes for practical use. The vignettes varied systematically: ranging from straight-forward classical presentations of anterior circulation stroke to more subtle symptoms suggestive of specific types of anterior or posterior circulation stroke; who observed the symptoms; and suspicion of the caller, in two vignettes they state thinking the patient was experiencing a stroke. Ease of recognition of vignettes was categorised by an expert panel consisting of five clinicians, two stroke survivors and six receptionists (not otherwise involved in the study). Role players will present vignettes either as the patient experiencing the symptoms or a relative (witness) calling to seek advice (Table 2).

**Table 2:** Vignette content

**ID**	**Brain territory of stroke**	**Who called**	Vignette details*	**Symptoms (no. FAST symptoms)**	**Perceived ease of recognition^**
1	Anterior	Adult child	I think my Mom’s having a stroke:	Facial droop (right side), right arm weakness and speech disturbance (3 FAST symptoms)	Easy
			• Her mouth is drooping		
			• Her speech is slurred		
			• She can’t use her right arm		
2	Anterior	Patient	I think I need to see the Doctor:	Arm weakness (1 FAST symptom)	Difficult
			• My arm’s gone all weak		
3	Posterior	Patient	I don’t know what to do:	Vomiting, vertigo and visual field defect (0 FAST symptoms)	Difficult
			• I keep throwing up		
			• I’m feverish		
			• I have double vision		
4	Anterior	Patient	I’m not sure what to do:	Facial droop (left side) (1 FAST symptom)	Moderately easy
			• When I look in the mirror my reflection looks funny		
5	Posterior	Patient	What shall I do I think I’m having a stroke?	Vomiting, vertigo and visual field defect (0 FAST symptoms)	Difficult
			• I’ve thrown up		
			• The room is spinning		
			• I have double vision		
6	Anterior	Adult child	Do you think my Father needs to see the Doctor?	Right arm weakness and speech disturbance (2 FAST symptoms)	Easy
			• He’s having difficulty speaking		
			• He can’t lift his arm up		
7	Anterior	Patient	I think I need to see the Doctor my Daughter tells me that:	Facial droop (left side) and arm weakness (2 FAST symptoms)	Moderately easy
			• My face is all droopy (left side)		
			• I keep dropping things		
8	Anterior	Adult child	Can I make an appointment for my Father?	Facial droop (right side) and speech disturbance (2 FAST symptoms)	Easy
			• His face is all lopsided (right side)		
			• He’s having trouble speaking		
9	Anterior	Adult child	I think my Mom needs to see the Doctor:	Speech disturbance (1 FAST symptom)	Moderately easy
			• Her speech is all slurred		
10	Anterior	Adult child	Shall I bring my Mother in to see the Doctor?	Facial droop (right side), right arm weakness and speech disturbance (3 FAST symptoms)	Easy
			• She can’t use her right arm		
			• She keeps dropping things		
			• Her face is really funny (right side)		
			• She’s talking a load of rubbish		

*If probed, symptoms were described as being ongoing and of having had a sudden onset within two hours of the telephone call.

^Probability of recognising symptoms was defined by an expert panel consisting of 5 clinicians, 6 receptionists (not otherwise involved in the study) and 2 stroke survivors.

Each role-player will be provided with detailed information regarding the context of each vignette and will be representative of the local population. In each vignette, the patient will only have had symptoms for 2 hours, therefore being eligible for thrombolysis but warranting immediate assessment by a specialist in order to remain within the optimum time period for such treatment. Dummy patient medical records will be generated for each simulated patient and these will be entered onto the clinical computer system of each participating practice by Practice Managers in order to minimise the potential for receptionist recognition of the unannounced simulated patient telephone call. It will not be possible to provide scripts for the role players as each call will be unique and dependent on receptionist responses. However, each telephone call will end with the role-player thanking the receptionist and informing them that the call was part of the RECEPTS study and that no further action is required.

The unannounced simulated patient telephone calls will be recorded for audit purposes although practices will have the option to opt out of recording. Recorded encounters may subsequently be used for linguistic analysis and the improvement of receptionist training. GP receptionist responses will be coded using an unannounced simulated patient telephone call data collection sheet, containing anticipated responses to the unannounced simulated patient telephone call (see Additional file 1).

The unannounced simulated patient telephone calls will be piloted in two practices prior to roll out, and findings will be integrated into the main study design. The aims of the pilot will be to: 1) assess the feasibility of generating simulated patient records within the practice computer system; 2) trial vignettes, each practice will receive five calls each, so that all ten vignettes will be trailed; 3) pilot the unannounced simulated patient telephone call data collection sheet; and 4) assess the systems for preventing potential adverse events, such as inappropriate engagement with Emergency Medical Services.

### Questionnaires

To assess knowledge and planned responses to stroke symptoms, reception staff from all practices within the study will receive a questionnaire, after the unannounced simulated patient telephone calls have been conducted at the practice. Questionnaires are commonly used to assess knowledge of stroke symptoms amongst patients and members of the public [33-37] and these will be adapted for use in this study. The questionnaire will gather reception staff demographic details, personal experience of stroke, job experience, training to recognise stroke, understanding of stroke symptoms and planned responses to a variety of symptoms, including some that are not normally associated with stroke. Questionnaires will be anonymous: a study ID which codes by GP will be recorded but not receptionist name. The validity of the questionnaire used in the present study will be assessed by piloting with a group of receptionists from one practice and follow up with a focus group to discuss interpretations of each question.

The effectiveness of the questionnaire at distinguishing between high and low levels of knowledge of stroke symptoms/responses will be assessed by comparing the questionnaire responses of healthcare professionals with differing levels of clinical training (receptionists, practice nurses and GPs). We will also examine the questionnaire responses of receptionists before and after a training session on stroke symptoms/responses to assess how effective it is at detecting changes in the stroke knowledge of participants from the target population. The results from individuals participating in the pilot will not be used in the main analysis.

### Answerphone message assessment

An assessment of GP answerphone messages will be carried out. Out of hours messages will be recorded and dialogue examined to see whether messages direct patients with specific symptoms to access other healthcare services, for example do they tell people with stroke symptoms to call the Emergency Medical Services.

### Qualitative

Qualitative methodology will be used to access study participants perceptions, understanding and attitudes towards handling stroke patients and GP receptionist training. The role of Primary Care reception staff in the referral of patients requiring urgent clinical intervention is currently poorly described; qualitative methods are especially applicable where a deeper understanding of contexts, interactions and processes is required to aid implementation of policy. Novel data will be generated about:

1) The views of Primary Care staff on the roles, skills and potential of reception staff with a particular focus on referral of patients.

2) Primary Care staffs’ awareness, understanding and experience of referral of patients by reception staff.

3) Opportunities for and barriers to effective referral of patients by reception staff and the Primary Care team.

4) Approaches and attitudes toward development of the role of reception staff within Primary Care.

5) Primary Care staffs’ perception of the training needs of reception staff and requirements to develop role in stroke patient referral.

Focus groups and interviews will be conducted in a setting determined by participant preference at a convenient location and time for participants and their Primary Care practice. All participants will give written informed consent. Sampling will be purposive to identify participants’ representative of a range of Primary Care staff, seniority and experience, practice size and location. Focus groups will be delineated by participants’ role. This approach will allow us to develop an explanation of the study objectives that encompasses the range of environments in which Primary Care is delivered.

Topic guides for in-depth interviews and focus groups will be informed by existing literature and simulated call results. It will be iteratively modified in response to emergent data as the study progresses. Topic guides will be discussed with and informed by the GP receptionist representative on the study steering group. All interviews and focus groups will be recorded, transcribed and checked for accuracy.

### Study outcome measures

The primary outcome measure will be reception staff response to unannounced simulated patient telephone calls. Primary Care staff will be graded on whether or not they correctly direct simulated patients for appropriate management. In addition, the associations between the nature of the telephone call, practice demographics and the subsequent response of the receptionist will be investigated.

The secondary outcomes will be:

1. Identifying GP receptionists’ knowledge of stroke via questionnaire.

2. An understanding of Primary Care staff perceptions of stroke, the role of reception staff and attitudes towards training, through a series of qualitative interviews and focus groups.

### Sample size

Sixty GPs will be recruited and data will be collected from a total of 600 unannounced simulated patient telephone calls. It is difficult to predict what proportion of patients would be expected to be correctly referred to hospital, as previous research is limited. A study by Mosley *et al.*[24], suggested that 55% of stroke patients were correctly referred by GP staff for immediate treatment. Given that the study assessment will be more rigorous, this proportion may be lower. Based on a conservative estimate that 50% of simulated patients will be correctly referred for immediate treatment and that each practice will receive 10 telephone calls over the study period (600 calls in total), an expected accuracy level would be to within ±4% (50% [95% CI 46% to 54%]). This calculation is based on the recruitment of one quarter of practices from the study area (60 out of 233 practices).

Questionnaire data will be collected from approximately 240 reception staff (assuming that each recruited practice has an average of four reception staff) and answerphone messages will be recorded in all participating practices (60 practices).

Qualitative data will be collected for up to 25 face-to-face in-depth interviews with a variety of Primary Care staff and up to 10 reception staff focus groups.

### Data analysis

#### Unannounced simulated patient telephone calls

Each unannounced simulated patient telephone call will be graded on the receptionist’s response, specifically whether they correctly direct patients to an emergency ambulance or transfer the patient’s call for immediate telephone assessment by a clinical member of the team. Logistic regression analysis will be used to investigate associations between correct referral of simulated patients, practice demographics (list size/number of receptionists) and the nature of unannounced simulated patient telephone call (e.g. perceived difficulty of detecting stroke, e.g. easy, moderately easy and difficult to recognise).

#### Questionnaire

Descriptive statistics will be used to describe the proportion of receptionists with each level of stroke symptom knowledge and the adequacy of planned responses to those symptoms. The association between stroke-related knowledge, planned responses and other demographic characteristics of GP receptionists (e.g. receptionist experience, age, gender, ethnicity, level of education and prior experience of stroke) will be examined using logistic regression analyses.

#### Answerphone message assessment

The content of messages and categories of referral advice, with a particular interest in the signs and symptoms they suggest require emergency action. Descriptive statistics will be used to outline content and suggested actions.

#### Qualitative

The qualitative component will follow the unannounced simulated telephone calls. The simulated call results will be fed into the conduct of the qualitative component, for example they may highlight areas that need further explanation to get a deeper picture [38]. Qualitative analysis will be performed using a framework approach [39]. This approach will allow the inclusion of *a priori* knowledge and specific objectives in the analysis and it is especially relevant to the development of practice based applications. Relevant theory will be considered. To maximise the efficacy and validity of the framework approach all members of the research team including GP receptionist representative will have the opportunity to contribute to its development. NVivo will be used to manage the data. Group comparisons will be made across the interview and focus group data sets to themes unique to particular staff groups in addition to those that are shared. Validation will be achieved using the constant comparison method and by triangulation between themes identified by study team members and where possible between study branches (e.g. questionnaire and results of unannounced simulated patient telephone call study). Study team members have a diverse background of disciplines and experience.

### Ethical considerations

Ethical approval has been obtained from the West of Scotland Research Ethics Service (reference: 12/WS/0259). Site specific R&D approval has been obtained from across Birmingham and Solihull Primary Care Providers.

## Discussion

The results of the RECEPTS study Primary Care are important for design of Primary Care services. Given the unusual nature of the proposed methodology, particularly the unannounced simulated patient telephone calls, it is important to acknowledge the potential problems involved in a study of this nature and how these might be avoided. Specifically:

1) There is a risk of reception staff recognising simulated patient records or unannounced simulated patient telephone calls. To reduce the chance of this happening: using multiple experienced role players, who have been trained by the Interactive Studies Unit, to conduct the telephone calls; having simulated patient records within the practice computer system so if the receptionist examines the clinical computer system for additional details the caller appears realistic; and conducting the calls over a period of time.

2) Concern that the unannounced simulated patient telephone calls will increase the burden of work for reception staff. To minimise this each practice will receive only 10 unannounced simulated patient telephone calls. Given the number of calls GPs receive per week (e.g. hundreds), an additional ten, with a maximum two minutes per call, over a three month period will not significantly impact their workload. GPs will be made aware of exactly how many unannounced simulated patient telephone calls to expect over the study period before they agree to participate.

3) The potential danger of inappropriate use of medical resources. To minimise this immediately following the unannounced simulated patient telephone call the GP receptionist, or whoever answered the call, will be informed that the telephone call was part of the study and thus no further action is needed.

4) Concern that receptionists will feel their performance is being examined. To reduce this our GP receptionist representative was involved in the design of study documentation, where the emphasis, particularly on the receptionist information cards is that this study is an opportunity to develop and improve training as well as patient care, not about testing. Furthermore, only whole study scores will be published – not individual practice or receptionist scores, so individuals will not be able to be criticised.

GP receptionists have a crucial role in patient referral, acting as gatekeepers to further care. It is important to gain an improved understanding of this role, how it plays out in the presentation of patients with serious conditions such as stroke and if there is a need for further training of reception staff in Primary Care. This study will provide this and also a valuable insight into the use of novel mixed methodologies which could have important implications for how health services are assessed in the future.

## Abbreviations

GP: General Practice; RECEPTS study: Receptionist rECognition and rEferral of Patients with Stroke Study; UK: United Kingdom.

## Competing interest

The Stroke Association (charity) is a non-financial partner in this research, their role is to assist with disseminating of the findings after analysis and write up have been completed. However within their portfolio they provide GP receptionist training sessions. They will not be involved with the analysis or interpretation of the data so should not influence the results. All authors declare that they have no conflicts of interests.

## Authors’ contributions

RM, JS, SS, had the original idea. RM, JS, SS, developed the protocol with JJ, EB and RJM. CW & JS advised on the simulation methodology and revised the protocol critically for intellectual content. All authors read and approved the final manuscript. RJM and RM gained the funding and RM is the guarantor.

## Pre-publication history

The pre-publication history for this paper can be accessed here:

http://www.biomedcentral.com/1471-2296/15/91/prepub

## Supplementary Material

Additional file 1 Example of an unannounced simulated patient telephone call data collection sheet. Click here for file
