# Supplementary material for: Receptionist rECognition and rEferral of PaTients with Stroke (RECEPTS) study - protocol of a mixed methods study
Source: BMC Fam Pract. 2014 May 12;15:91. doi: 10.1186/1471-2296-15-91 (PMC4030067; doi:10.1186/1471-2296-15-91)
Supplement: Additional file 1 — Example of an unannounced simulated patient telephone call data collection sheet. [file 1471-2296-15-91-S1.docx]

Additional file 1: Example of an unannounced simulated patient telephone call data collection sheet

**
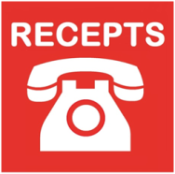
VIGNETTE**

**Version 6** **dated 05/03/2013**

**Receptionist recognition and referral of patients with stroke (RECEPTS) study**

Intro blurb: *I think my Mum's having a stroke…..*

*Her mouth is drooping*

*Her speech is slurred*

*She can't use her right arm*

| Vignette ID | XXX |
| --- | --- |
| **Your role** | **Adult child** |
| Practice details |  |
| Practice name | XXX |
| Practice phone no. | XXX |
| Practice address | XX  XX |
| Name of doctor patient is registered with | XXX |
| Name of Practice Manager | XXX |
| Patient details |  |
| Name | XXX |
| Age | XX |
| Date of birth | XX/XX/19XX |
| Address | XX  XX  XX  XX |
| Telephone Number | XX |
| Time since symptom onset | 2 hours |

***Thank you. This phone call was part of the University of Birmingham RECEPTS study and you don’t need to do anything else today.***

*(Please tick box when you have said this to the receptionist)*

**
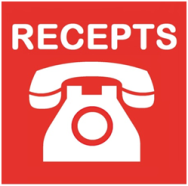
** **VIGNETTE DATA COLLECTION FORM**

**Version 5** **dated 05/02/2013**

**Receptionist recognition and referral of patients with stroke (RECEPTS) study**

| Vignette ID |  |
| --- | --- |
| Date of call |  |

| Time of first call |  |
| --- | --- |
| No. calls made |  |

Action taken by receptionist (please tick ONE):

| Advised to call **999** |  |
| --- | --- |
| Put **through to GP** immediately |  |
| Advised to attend **A&E** |  |
| Advised to attend **out of hours service** |  |
| Advised to attend **GP practice immediately** |  |
| Advised that GP will **call patient back later** that same day |  |
| Advised to **attend practice** later that **same day** |  |
| Offered/given an **appointment** with GP for the **next day** |  |
| Offered/given and **appointment** with GP **more than one day away** |  |
| Other (please specify) |  |

Reaction of receptionist when fake call revealed to them:

|  |
| --- |

Other notes (optional)

|  |
| --- |

Name of role player: _____________________
